# Functionalization of graphene using deep eutectic solvents

**DOI:** 10.1186/s11671-015-1004-2

**Published:** 2015-08-12

**Authors:** Maan Hayyan, Ali Abo-Hamad, Mohammed AbdulHakim AlSaadi, Mohd Ali Hashim

**Affiliations:** University of Malaya Centre for Ionic Liquids (UMCiL), University of Malaya, Kuala Lumpur, 50603 Malaysia; Department of Civil Engineering, University of Malaya, Kuala Lumpur, 50603 Malaysia; Department of Chemical Engineering, University of Malaya, Kuala Lumpur, 50603 Malaysia; Nanotechnology & Catalysis Research Centre (NANOCAT), University of Malaya, Kuala Lumpur, 50603 Malaysia

**Keywords:** Deep eutectic solvents, Ionic liquids, Functionalization, Graphene, Dispersion stability, Nanotechnology

## Abstract

Deep eutectic solvents (DESs) have received attention in various applications because of their distinctive properties. In this work, DESs were used as functionalizing agents for graphene due to their potential to introduce new functional groups and cause other surface modifications. Eighteen different types of ammonium- and phosphonium-salt-based DESs were prepared and characterized by FTIR. The graphene was characterized by FTIR, STA, Raman spectroscopy, XRD, SEM, and TEM. Additional experiments were performed to study the dispersion behavior of the functionalized graphene in different solvents. The DESs exhibited both reduction and functionalization effects on DES-treated graphene. Dispersion stability was investigated and then characterized by UV–vis spectroscopy and zeta potential. DES-modified graphene can be used in many applications, such as drug delivery, wastewater treatment, catalysts, composite materials, nanofluids, and biosensors. To the best of our knowledge, this is the first investigation on the use of DESs for graphene functionalization.

**Electronic supplementary material:**

The online version of this article (doi:10.1186/s11671-015-1004-2) contains supplementary material, which is available to authorized users.

## Background

Graphene is a carbon nanomaterial (CNM) that can be used for numerous applications, and it has attracted the intense interest of researchers. It is comprised of sp^2^-hybridized carbon atoms packed in a hexagonal network structure to form a flat, two-dimensional sheet [1]. Graphene is easy to functionalize, and it has unique physicochemical properties, such as excellent thermal and electrical conductivities, high surface area, and good mechanical strength [2]. As a result, graphene is applicable in chemical and biochemical sensors, electronics, and energy storage devices [3, 4]. However, the agglomeration of nano-sized carbon is one of the primary obstacles that limits the applications of such devices; therefore, this is an important problem that must be solved. The effects of π-π adhesion and van der Waals interactions generally are responsible for this tendency. These effects may sometimes cause the restacking of graphene sheets to form graphite [5, 6]. Thus, surface modification can be introduced to reduce agglomeration and allow the implementation of the inherent properties of graphene.

Functionalization is a process that includes the addition of new functional groups to the surface of the carbon through a chemical or physical attachment. According to this connection, functionalization is classified into two types, i.e., covalent and non-covalent functionalization [7–9]. With hydrophobic or hydrophilic functional groups on the surface of the carbon, dispersibility of CNMs can be improved effectively in different types of solvents. This is caused by reducing the strength of polar-polar interactions and/or the splitting of bulky structures [10]. A graphene oxide (GO) nanosheet can be produced by treating graphite with strong oxidant acids using Hummers’ method [8, 11].Then, the product is susceptible to covalent and/or non-covalent treatment with different materials. However, both treatments have their respective benefits for applications. Non-covalent contact between two films of GO and porphyrin was reported as highly significant for producing a photocurrent in a photoelectrochemical cell [12]. The composite film was fabricated using a layer-by-layer method, prior to vapour reduction of the GO-porphyrin films. The advantages of this system are due to the π-π and electrostatic interactions between the GO sheets and the porphyrin molecules. In another study, GO was used with two types of water-soluble polythiophenes to prepare composite films with enhanced photoresponse [13]. In this case, a chemical reduction and π-stacking took place in one of the prepared GO/polymer suspensions followed by a layer-by-layer treatment with the second polymer solution to fabricate the photoactive film. Similarly, GO and poly(3-hexylthiophene) were used to fabricate a “dual-fluorescent material” with tunable photoluminescent properties [14]. The final product was useful in sensing amine-based pollutants due to its ratiometric fluorescence response. Apart from the sensing application, the same composite has been used in the design of photo-controlled switches [15]. An improved photo-thermal effect was recorded for the composite because of the photo-induced energy transfer between the graphene and the polythiophene.

Generally, the most common conventional materials used for functionalization are strong inorganic acids, such as sulfuric acid, nitric acid, and mixtures of the two acids. Highly-volatile organic solvents also have been used in this respect. However, due to the associated risks and precautions of using such chemicals, it is highly essential to develop safer and more eco-friendly alternatives [16].

In the past decade, there was increased interest in ionic liquids (ILs) as new "green" solvents because of their unique physicochemical properties [17, 18]. Zhao *et al*. used an IL, namely 1-(3-aminopropyl)-3-methylimidazolium bromide, as a functionalizing agent to modify graphene oxides [19]. The resulting carbon was used to construct a novel, carbon-based sensor for the determination of sunset yellow in soft drinks. A similar functionalization process was conducted by Chai and co-workers [20] by using the same materials. The carbon that was obtained was used, along with hollow AuPd nanoparticles, as an effective catalyst support for the electro-oxidation of formic acid. However, there are some limitations to the use of ILs, such as the high cost of synthesis, their toxicity, corrosivity, and non-biodegradability.

In 2003, special analogues of ILs were introduced and defined as deep eutectic solvents (DESs) [21]. These solvents generally are based on mixtures of a quaternary ammonium or phosphonium salts and an uncharged hydrogen bond donor (HBD), such as an amide, acid, or alcohol. The main feature of DESs is that the melting point of the mixture is lower than that of any of its individual components [22]. In general, DESs are simple to synthesize, and the synthesis cost is low. In addition, DESs are expected to be non-toxic or less toxic than ILs and to have better biocompatibility than ILs [23–25]. Hence, there are numerous potential applications of DESs in nanotechnology [26].

In this study, selected ammonium- and phosphonium-based DESs were used as novel functionalizing agents for graphene. In addition, the DESs were compared based on the structure of the graphene and the improvement in dispersibility. Changes on the structure of the surface were identified by FTIR, Raman spectroscopy, XRD, STA, and TEM. The dispersibility and the stability of the suspension of the DES-functionalized graphene were investigated and characterized using UV–vis spectroscopy and zeta potential analysis.

## Methods

### Materials

Methyltriphenyl-phosphonium bromide (purity ≥98%), N,N-diethylethanolammonium chloride (purity ≥98%), sucrose, urea (purity ~99.5%), ethanol, (purity ≥99.9%), ethylene glycol (purity 99%), diethylene glycol (purity 99%), and triethylene glycol (purity 99%) were purchased from Merck (Darmstadt, Germany). Choline chloride (2-hydroxyethyl-trimethylammonium) (purity ≥98%) and malonic acid (purity 99%) were provided by Sigma-Aldrich. Anhydrous D(+)-glucose, D(−)-fructose (purity >98.0%) and glycerol (purity 99.8%) were obtained from R&M Chemicals, and potassium permanganate was obtained from Univar (purity 99%).

Sixty-nanometer flakes of graphene nanopowder (AO-4) grade were obtained from Graphene Supermarket (USA) with the following specifications: purity 98.5%, lateral particle size ~ 3–7 microns, 60 nm average flake thickness, and specific surface area <15 m^2^/g.

Table 1 provides a list of the chemicals used for the preparation of DES.

**Table 1:** List of chemicals used with their formulae and abbreviations

Name of material	Abbr.	Formula	Name of material	Abbr.	Formula
Choline chloride	ChCl		Urea	U	
*N*,*N*-Diethylethanolammonium chloride	N,N		Malonic acid	MA	
Methyltriphenyl-phosphonium bromide	MPB		d-(+)-Glucose	Glu	
Glycerol	Gly		d-(−)-Fructose	Fru	
Ethylene glycol	EG		Sucrose	Suc	
Diethylene glycol	DEG		Water	W	
Triethylene glycol	TEG				

### DES synthesis

All solid materials were dried at 60 °C under vacuum before they were used. All of the DESs listed in Table 1 were prepared by following the Abbott *et al*. method [21, 27]. Salts and HBDs were mixed according to the given molar ratio using a jacketed vessel accompanied by mechanical or magnetic stirring for liquid or solid HBDs, respectively). The temperature was set to a minimum of 70 °C during the synthesis of the DESs, which took no more than 3 h at atmospheric pressure and under tight moisture control. The resulting transparent, homogenous liquids were transferred to well-sealed and dark vials.

Fresh DES samples were used for all experiments and analyses to avoid any possible contamination because of humidity or structural changes over time. Eighteen types of DESs were prepared from different ammonium and phosphonium salts and different HBDs. Table 2 provides the details concerning the DES components, molar ratios, and abbreviations.

**Table 2:** List of DESs prepared with molar ratios and abbreviations

DES	Molar Ratio (Salt:HBD)	Abbreviation
ChCl:Gly	1:2	DES 1
ChCl:EG	1:2	DES 2
ChCl:DEG	1:2	DES 3
ChCl:TEG	1:2	DES 4
ChCl:U	1:2	DES 5
N,N:Gly	1:2	DES 6
N,N:EG	1:2	DES 7
N,N:DEG	1:2	DES 8
N,N:TEG	1:2	DES 9
MPB:Gly	1:3	DES 10
MPB:EG	1:3	DES 11
MPB:DEG	1:3	DES 12
MPB:TEG	1:3	DES 13
Glu:ChCl:W	2:5:5	DES 14
ChCl:Fru:W	5:2:5	DES 15
Suc:ChCl:W	1:4:4	DES 16
ChCl:Gly:W	1:2:1	DES 17
ChCl:MA	1:1	DES 18

### General procedure for the pretreatment of graphene

Graphene nanopowder flakes were dried at 100 °C under a vacuum for 3 h to eliminate any water that was present on the surface of the carbon. A 1-M solution of KMnO_4_ was prepared at 70 °C for ease of solvation. Graphene was oxidized by the following procedures: 1) 200 mg of graphene were mixed with 7 ml of the previous permanganate solution inside a 20-ml glass vial; 2) the mixture was subjected to ultrasound waves using an ultrasonic bath at 70 °C for 3 h; 3) graphene was collected by a filtration process under vacuum using filter paper with a pore size of 0.45 μm; 4) the graphene was washed several times with 0.01M HCl and distillated water until a clear, transparent product was obtained; and 5) the collected cake was dried for 3 h at 100 °C.

### Graphene functionalization process

In a procedure similar to the previous chemical treatment of the graphene flakes, functionalization was conducted ultrasonically in a 20-ml glass vial that contained 200 mg of oxidized graphene and 7 ml of DES. The suspension was sonicated for 3 h at 60 °C and then filtered under vacuum. The retained carbon was washed with water until a transparent effluent was produced that had the same pH as the influent. The experiments included 18 different DESs, and, for comparison, one experiment was conducted with the pristine graphene without prior treatment with KMnO_4_. Graphene was collected and dried in air at 100 °C for at least 3 h and then transferred to a clean-air desiccator where it was allowed to cool to room temperature. Then, the graphene samples were ready for characterization.

### Instruments and measurements

The FTIR measurements of the carbon samples before and after the treatment were conducted using a Perkin Elmer FTIR spectrometer with a range of 450–4000 wavenumbers and a four-time scanning repetition. All DESs and their individual components were analyzed under the same conditions. Thermogravimetric analysis (TGA) and Differential Thermogravimetry (DTG) were performed simultaneously for all samples using the Simultaneous Thermal Analyzer (STA-6000, PerkinElmer). The tests were performed in an N_2_ environment and at a heating rate of 5 °C/min. Raman spectroscopy (Renishaw System 2000 Raman Spectrometer) and X-ray diffraction tests (XRD) also were performed in this study.

In order to obtain a fair idea of the successful modification of graphene, a series of dispersibility tests was conducted using solvents with different polarities, e.g., distillated water, acetone, and n-hexane [28]. First, graphene was dispersed in the solvent at a concentration of 1 mg/ml, and the mixture was sonicated for 10 min and then allowed to settle for 24 h. Photos of the dispersion were taken before and after the settling, and the dispersibility was evaluated based on the particles of graphene that were precipitated [29].

UV–vis spectroscopy was conducted using PerkinElmer-Lambda [30] to characterize the dispersion behavior of pristine graphene and oxidized graphene in a water medium. A suspension of 0.5 mg/ml was prepared and sonicated for 1 h before the analysis. Additional characterization of the treated and untreated samples was conducted for zeta potential and particle size using a Zetasizer (Malvern, UK).

## Results and discussion

### DES FTIR spectra

**Fig. 1:**
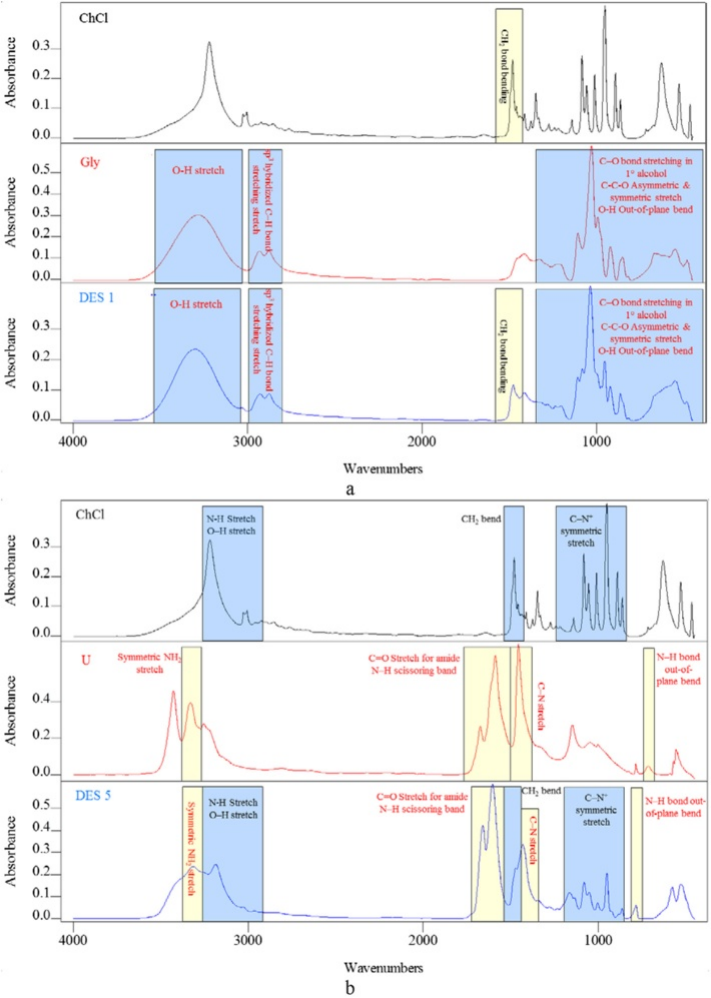
FTIR spectra for DES 1 (**a**) and DES 5 (**b**) along with their individual components

Before identifying the structural changes on the surface of the carbon, a preliminary investigation was conducted to recognize the functional groups of DES as functionalizing agents. Therefore, FTIR spectra were studied for all of the selected DESs and their individual components. The spectrum of each DES, which is a combination of two or more components, was interpreted based on the well-known and recognized peaks of DES components. Figure 1(a) and (b) show the FTIR spectra for DES 1 and DES 5 along with their individual components, i.e., ChCl, Gly for DES 1, and ChCl, U for DES 5.

The details of the FTIR spectra are provided in Additional file 1: Figures S1-S38, and they are illustrated in Additional file 2: Tables S1-S18 in the Supplementary Appendix. For each DES, a comparison was made between the DES spectrum and its individual components. Each table relates to a type of DES and involves the peaks in the spectrum of the DES and in its individual components. The peaks of the DES spectra may be identical to those of salt or HBD. However, some shifting was noticed in some peaks, as shown in Fig. 1 and Additional file 1: Figures S1-S18.

Additional file 3: Table S19 indicates the peaks that were detected for each DES and the matching functional group. The origins of the functional groups, which can be either from the salt or the HBD, also are provided in Additional file 3: Table S19. For the DESs prepared from liquid HBDs, the FTIR spectra tended to simulate the spectra of their liquid components with only a few differences, which were attributed to the contribution of the salt. References used to interpret the FTIR spectra of the DESs and their individual components are available in previous studies [30–35].

### Glycolic-based DESs (1, 2, 3, 4, 6, 7, 8, 9, 10, 11, 12, and 13)

Table 3 lists the functional groups extracted from the glycolic HBDs after the formation of the DESs. These groups are in accordance with all glycolic compounds, i.e., G, EG, DEG, and TEG, although each one has some unique functional groups. The O-H bond was found in the glycolic-based DESs and in all other DESs used in this study. Stretching vibration, which was detected in all of the DESs, was the most common vibration for this functional group, while in-plane bending vibration was detected in only a few types, as shown in Table 3. However, the OH group may reflect the intentional or unintentional presence of H_2_O from the raw materials that were used, since all of the HBDs had this functional group. The C–H, C–O, and C-C-O functional groups were identified in all glycolic-based DESs. They seemed to be the dominant groups that existed when the four glycols were involved.

**Table 3:** Functional groups coming from glycolic HBDs after DES formation with different salts

Functional group	Involving DES
O-H (stretching)	1,2,3,4,6,7,8,9,10,11,12,13 (all)
sp^3^ hybridized C–H (stretching)	All
C–O (stretching) in 1° alcohol	All
C-C-O (asymmetric stretching)	All
C-C-O (symmetric stretching)	All
O-H (Out-of-plane bending)	All except 13
C–O–H (bending)	2,3,6,7,8,10,11,12
O-H (In-plane bending)	3,11,12,13
CH_2_ (bending)	6,7,8,9,10,11,12,13

#### ChCl-based DESs (1, 2, 3, 4, 5, 14, 15, 16, 17, and 18)

Among all of the functional groups that existed in ChCl, only five of them can co-exist after the formation of the DESs (Table 4). The prominent group detected in all ChCl-based DESs was CH_2_. However, the remaining four groups were found in minute quantities in some types of DESs. Having urea as part of the mixture enabled the appearance of N-H and C–N^+^ bonds from a ChCl source. Thus, three ChCl-based functional groups out of four can be detected with the presence of U as an HBD.

**Table 4:** Functional groups coming from ChCl salt after DES formation

Functional group	Involving DES
CH_2_ (bending)	All
N-H (stretching)	2,5,18
C–N^+^ (symmetric stretching)	3,4,5
CH_3_ (asymmetric stretching)	14,16

#### N,N based-DESs (6, 7, 8, and 9)

Only three functional groups were identified in the N,N-based DESs (Table 5). C-H groups were observed in all N,N-based DESs prepared from glycolic HBDs. The type of glycolic HBD tended to have a role in determining the salt contributions by controlling the functional groups that were being identified.

**Table 5:** Functional groups coming from N,N salt after DES formation

Functional group	Involving DES
C-H (deformation)	All
N-H^+^ (stretching) of quaternary ammonium	7,9
C–N^+^ (symmetric stretching)	7,8

#### MPB-based DESs (10, 11, 12, and 13)

The aromatic ring in MPB was clearly identified in MPB-based DESs as =C-H and C=C groups. The bond between this ring and the phosphorus atom also was found in all of the DESs based on MPB salt as was the case in the salt itself. However, the P-CH_3_ functional group was detected in the DEG-based DESs, as illustrated in Table 6.

**Table 6:** Functional groups coming from MPB salt after DES formation

Functional group	Involving DES
=C-H and ring C=C (stretching)	10,11,12,13 (all)
P-Phenyl (stretching)	All
P-CH_3_ (asymmetrically CH_3_ deformation)	12
P-CH_3_ (C-H rocking)	12

### Effects of the pretreatment of graphene

**Fig. 2:**
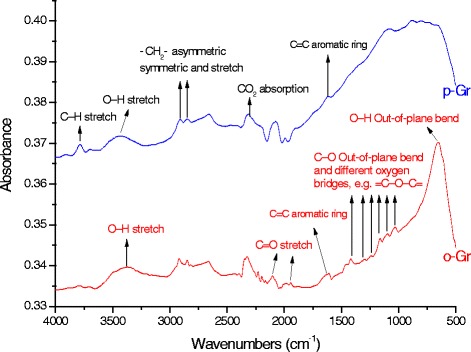
FTIR spectra of pristine graphene (p-Gr) and oxidized graphene (o-Gr)

According to the FTIR results shown in Fig. 2, the spectrum of oxidized graphene (o-Gr) was rich in peaks of oxygen-containing functional groups. The spectrum showed the stretching vibration of the C=O band between 1700–2200 cm^-1^ and other oxygen bridges, such as C–O–C, C–O–H, and C–O, between 1463–1020 cm^-1^ [36, 37], which was not the case the pristine graphene (p-Gr). The bands at wavenumbers 3392 cm^-1^ and 657 cm^-1^ might correspond to the O–H bond with stretching and bending vibrational modes [9]. However, in the p-Gr spectrum (blue line), the prominently-detected peaks corresponded to =CH_2_ asymmetric and symmetric stretching at 2915 cm^-1^ and 2850 cm^-1^ [38] and at 3783 cm^-1^ and 672 cm^-1^, which were expected to be for the stretching and bending vibrations of the C–H bond, respectively. The C=C from unoxidized sp^2^ CC bonds was found at 1618 cm^-1^ [39, 40], while the peak at 2300 cm^-1^ was caused by the absorption of CO_2_ from the air [41]. These prominent groups in p-Gr have a hydrophobic nature, which determines the dispersibility behavior in aqueous and non-aqueous solutions.

**Fig. 3:**
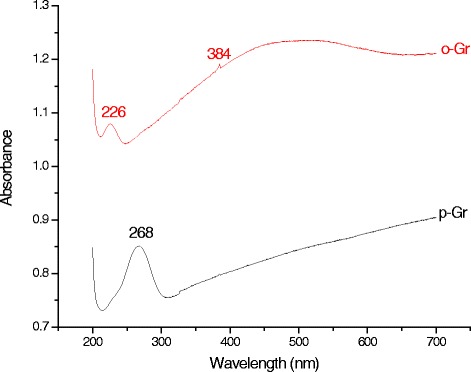
UV–vis spectra of pristine graphene (p-Gr) and oxidized graphene (o-Gr) dispersed in distillated water

The p-Gr and o-Gr were dispersed in water and scanned spectrophotometrically in the wavelength range of 200–700 nm. Figure 3 shows that the dispersion of pristine graphene had strong UV absorption at 268 nm, which can be ascribed to the π–π* transition of the C=C bond. After treatment with KMnO_4_, the peak shifted to 226 nm, and a shoulder peak appeared at 384 nm, indicating the n–π^*^ transition of carboxyl groups [29, 42, 43].

**Fig. 4:**
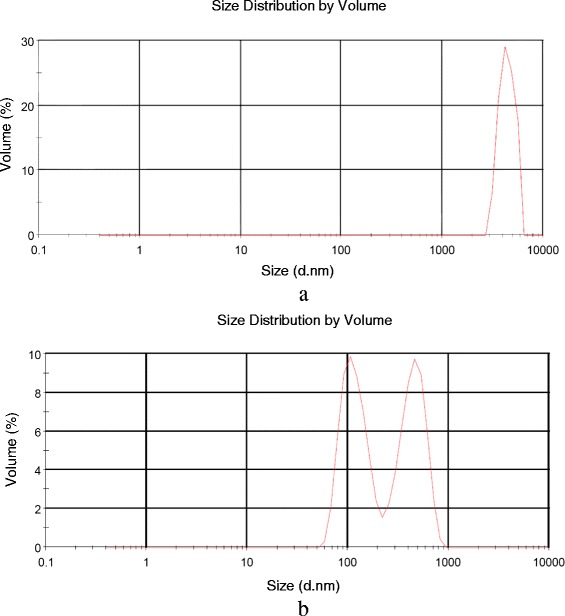
Size distribution of pristine graphene **a**, Z-Average 3.364 μM and oxidized graphene **b**, Z-Average 0.4773 μM

The aqueous dispersions of p-Gr and o-Gr were characterized further by dynamic light scattering (DLS). The study was conducted using the standard model of a spherical particle (Fig. 4). Graphene particles were obtained by the prolonged sonication of p-Gr and o-Gr in water, and the average sizes were 3.364 μm and 0.4773 μm, respectively. However, while graphene particles are not spherical, the model can be used to identify relative changes in size after the pretreatment. The results corresponded with the specifications of the p-Gr that was used (i.e., a particle size between 3–7 μm) and indicated a significant decrease in the particle size by a ratio of 1/7 after treatment with KMnO_4_. Further information about the impact of pretreatment with graphene is discussed in the Raman spectroscopy analysis, XRD test, and STA sections.

### FTIR of DES-modified graphene

**Fig. 5:**
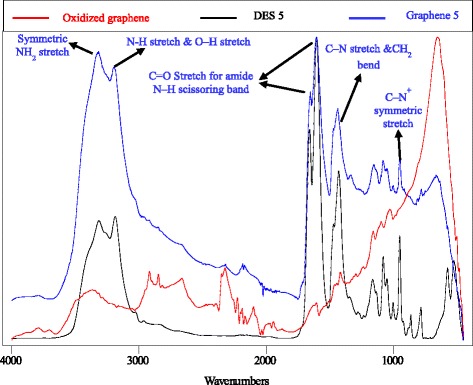
FTIR spectra for DES 5, oxidized graphene and functionalized graphene

FTIR analyses for a series of DES-modified graphenes were conducted to characterize the functionalization process. Figure 5 shows the FTIR spectra of oxidized graphene, DES 5, and DES 5-functionalized graphene (graphene 5) as examples. However, all FTIR spectra of treated and untreated graphene samples are provided individually in Additional file 1: Figures S19-S39. After the DES treatment, the results showed that various functional groups appeared. These bonds can be tuned for a required purpose by modifying different DESs. Table 7 illustrates the cases in which the changes on the graphene are detectable by FTIR analysis. However, not all DESs produced new functional group allocations on the surface of the carbon. DESs 7, 11, and 18 influenced the spectra on which the peaks related to impurities or oxygen bonds totally disappeared, indicating the reduction effect. It was clearly noted that the minimum number of peaks occurred for graphene treated with DES 18. This sample represented the highest purity sample with no intense peaks in its spectrum. The high acidity of this DES (pH = 0.14) was responsible for cleaning the surface of the graphene. Conversely, DES 5 was the most affecting agent among the DESs, and five new peaks were detected after treatment, all of which corresponded to peaks in the DES spectrum. The bands between 3326–3186 cm^-1^ represented −NH_2_ and −NH− stretching vibrations, whereas the in-plane stretching of –NH was evident at ~1605 cm^-1^ [44, 45].

**Table 7:** New detected peaks in FTIR spectra of some graphene samples after DES treatment with the expected matching functional groups

Graphene sample	New allocated peaks	Expected functional groups
1	3356	O–H (stretching)/asymmetric NH_2_ (stretching)
	1930	CH_2_ (bending)/C=O
	1362	C–O–H (bending)/C–O (stretching), alcohol, ester, ether, carboxylic acid
	1078	C-C-O (asymmetric stretching)
	506	O-H (Out-of-plane bending)/C–H (bending)
3	3794	O–H (stretching)/– OH (unbonded)
	2171	sp^3^ hybridized C–H (stretching)
	1980	CH_2_ (bending)
	1937	sp^3^ hybridized C–H (stretching)/C–O (stretching), alcohol, ester, ether, carboxylic acid
	1330	O-H (In-plane bending)/N–H (bending)
	1248	C–O–H (bending)/C–O (stretching), alcohol, ester, ether, carboxylic acid
5	3322	NH_2_ (symmetric/asymmetric stretching)
	3199	O–H (stretching)/–CH_2_ (antisymmetric stretching)
	1606	1° amide N–H scissoring band
	1444	C–N (stretching)
	950	C–N^+^ (symmetric stretching)/N–O (in-plane bond)
6	3209	sp^3^ hybridized C–H (stretching)
7	1100-1150	C–O–H (bending)/C–O (stretching), alcohol, ester, ether, carboxylic acid
8	531 (shoulder peak)	C–N^+^ (symmetric stretching)
9	1240	C–O–H (bending)
	2476	N-H^+^ (stretching) of quaternary ammonium
12	1319	C–O–H (bending)
	1255	C–O–H (bending)/C–O (stretching), alcohol, ester, ether, carboxylic acid
13	2164	=C-H and ring C=C (stretching)
	1323-1255	C–O–H (bending)/C–O (stretching), alcohol, ester, ether, carboxylic acid
14	2661	N-CH_3_ (symmetric stretching)/–CH_2_ (antisymmetric stretching)
	2203	CH_2_ (bending)/–CH_2_ (symmetric stretching)
	1323	CO (stretching) + CCH (stretching) + ring of pyranose (antisymmetric stretching)/N–H (bending)
	1255	CO (stretching) + CCH (stretching) + ring of pyranose (antisymmetric stretching)/C–O (stretching), alcohol, ester, ether, carboxylic acid
15	3353	O-H (stretching)/asymmetric NH_2_ stretch
	1930	CH_2_ bond bending vibration/C–O (stretching), alcohol, ester, ether, carboxylic acid
	1330	CO (stretching) + CCH (stretching) + ring of pyranose (antisymmetric stretching)
	1255	CO (stretching) + CCH (stretching) + ring of pyranose (antisymmetric stretching)/C–O (stretching), alcohol, ester, ether, carboxylic acid
	1078	C–N^+^ (symmetric stretching)
	479	CCO (in-plane bending) + CCH (in-plane bending)/C–H (bending)
16	2161	CH_2_ (bending)/– CH_2_ (symmetric stretching)
	1987	CH (symmetric stretching) of C_2_/C–O (stretching)
	873 (shoulder peak)	C–N^+^ (symmetric stretching)
	503 (shoulder peak)	CH (in-plane bending) + CC (stretching)+ CC (in-plane bending)
17	2027	CH_2_ (bending)

**Fig. 6:**
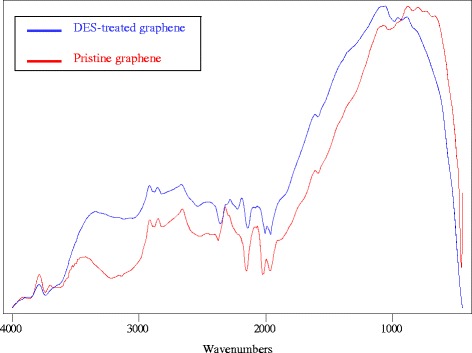
FTIR spectra for pristine graphene and DES 5-modified graphene without any pretreatment

Given the above observations, it is apparent that at any rate, the treatment of graphene with KMnO_4_ accounted for the changes in the functional groups obtained from DES on the carbon surface. To prove this, an experiment was conducted in which pristine graphene was treated directly with DES 5. The FTIR spectrum of the sample that was obtained was identical to the spectrum for untreated graphene (Fig. 6). Cleaning the surface and/or causing some deformation of the surface of the carbon may have occurred after the treatment with KMnO_4_. These changes are the keys for surface activation because they make the surface capable of absorbing, adsorbing or desorbing functional groups. The covalent functionalization of graphene can take place in two main modes, i.e., 1) covalent bonds between free radicals or dienophiles and C=C in the skeleton of pristine graphene, and 2) covalent bonds between an organic group and the oxygen-containing functional groups in graphene oxide [7]. Thus, the covalent functionalization reaction occurs as a result of forming covalent bonds with oxygen functional groups in o-Gr. Later, Raman spectroscopy, thermogravimetric analysis, and zeta potential are discussed to illustrate these points further.

A convenient comparison between all carbon spectra was conducted, and the results are summarized in Additional file 4: Table S20 in the Supplementary Appendix. Note that graphene sheets rich in oxygen-containing functional groups can be applicable in wastewater treatment, e.g., for the removal of heavy metals [46]. It has been suggested that strong surface complexes form between positive metallic ions and oxygen-containing functional groups. Thus, since most of the modified samples had oxygen on their surfaces, they may be useful in removing heavy metals from wastewater and other waste streams.

### Thermal stability

In order to confirm the presence of oxygen-containing functional groups and other groups, the thermal stabilities of the graphene samples were examined using TGA and DTG.

In the following sections, samples of DES-functionalized graphene oxide are divided into four groups to facilitate their comparisons. The samples functionalized by DES 1 to 5, which were based on ChCl salt (Gr 1, 2, 3, 4, and 5), are represented in Group 1. The samples modified by DES 6 to 9, based on N,N salt (Gr 6, 7, 8, 9), are in Group 2. Gr 10, 11, 12, and 13, modified by MPB-based DES, are in Group 3. The rest of the sample were treated with natural DES 14–18 (provided in a previous study [47]), and Gr 14, 15, 16, 17, and 18 make up Group 4.

**Fig. 7:**
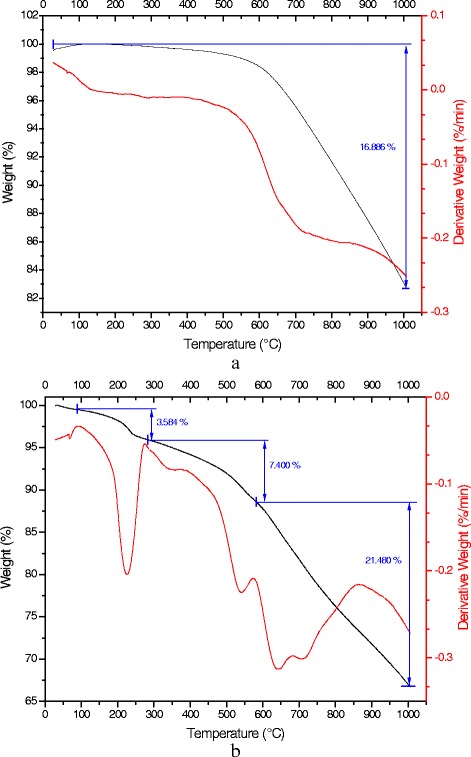
Thermogravimetry (TGA) and differential thermogravimetry (DTG) curves of pristine (**a**) and oxidized graphene (**b**)

The tests were conducted in a nitrogen atmosphere. Figure 7 shows TGA and DTG curves for p-Gr and o-Gr. The figure shows that p-Gr had better thermal stability than o-Gr and the DES-functionalized graphene samples. Total weight loss was 17% for p-Gr, which started above 500 °C. This loss corresponded to the destruction of the carbon skeleton (carbonyl/double bond) of graphene [48]. The DTG curves provided the variation in weight with time (dW/dT) and assisted in identifying the degradation steps of graphene. The peaks in the DTG curves reflect the temperature of the maximum reactive velocity. The TGA and DTG curves for DES-functionalized graphene are shown in Additional file 1: Figures S39-S56 in the Supplementary Appendix.

Based on their TGA and DTG curves, nearly all of the oxidized and DES-functionalized graphene samples were found to have three degradation steps. Therefore, Table 8 provides a general comparison of the samples to show the differences in their thermal behaviors and to confirm the addition and/or elimination of functional groups. The effect of water content was negligible since it did not exceed 0.2% of the total weight because the samples had been dried previously. The first step of degradation in o-Gr and in all of the DES-functionalized graphene samples occurred in the range of 90–280 °C. The next step began above 280 °C and continued until the temperature reached ~550 °C ± 30 °C. Then, the final degradation began until the end of the test at 1000 °C. The first and second degradations were caused by the loss of oxygen-containing functional groups or any other groups that were present [49]. The last step was normally caused by the decomposition of the remaining unstable carbon, as evidenced in previous studies for different graphene composites [50, 51].

**Table 8:** Comparative data on graphene thermal behavior using TGA-DTG analyses

Sample	1st degradation		2nd degradation		3rd degradation		Total weight loss (%)	Remaining weight (%)
	Weight loss (%)	T_max_^a^ (°C)	Weight loss (%)	T_max_^a^ (°C)	Weight loss (%)	T_max_^a^ (°C)		
p-Gr	0.000	-	0.000	-	16.886	>500	16.886	83.114
o-Gr	3.584	225	7.400	539	21.480	643	32.464	67.536
Gr-1	5.029	176	8.631	533	24.154	698	37.814	62.186
Gr-2	4.160	191	7.287	551	22.253	726	33.700	66.300
Gr-3	3.505	198	7.741	531	19.976	713	31.222	68.778
Gr-4	3.495	202	7.156	550	23.457	728	34.108	65.892
Gr-5	2.712	203	2.829	546	18.801	722	24.342	75.658
Gr-6	4.799	167	4.458	487	22.889	710	32.146	67.854
Gr-7	3.387	179	4.376	510	22.062	711	29.825	70.175
Gr-8	3.786	186	4.789	526	23.732	700	32.307	67.693
Gr-9	3.399	188	4.177	509	22.108	722	29.684	70.316
Gr-10	4.727	185	6.098	543	24.493	719	35.318	64.682
Gr-11	4.192	194	5.920	533	17.356	705	27.468	72.532
Gr-12	4.505	194	5.953	524	19.379	713	29.837	70.163
Gr-13	4.815	195	4.992	528	25.581	721	35.388	64.612
Gr-14	4.808	199	5.395	499	17.249	698	27.452	72.548
Gr-15	2.124	219	-	-	28.261	720	30.385	69.615
Gr-16	3.225	203	6.187	547	17.779	713	27.191	72.809
Gr-17	4.111	189	7.627	554	22.929	722	34.667	65.333
Gr-18	2.767	211	2.900	322	23.881	729	29.548	70.452

^a^Temperature at maximum rate of mass loss

The rate of maximum weight loss in o-Gr within the temperature range of 90–280 °C occurred at 225 °C; however, the rate decreased after DES functionalization. The second degradation was observed in various temperature ranges for the different groups of graphene samples. In Group 1 (graphene functionalized by ChCl-based DES), this range was 280–580 °C, while it was narrower in the other groups. The presence of oxygen-containing functional groups accounted for o-Gr’s having less thermal stability than p-Gr. Moreover, some samples showed less thermal stability than o-Gr due to the functionalization effect and the introduction of new functional groups, such as in Gr 1, 2, 4, 10, 13, and 17. By contrast, the total weight loss was decreased noticeably in most samples, indicating the deoxidation effect of DES as a result of the elimination of functional groups and/or their replacement with other groups from the DES source.

In the four groups that were studied, it was observed that the graphene samples treated with Gly-containing DESs had higher weight loss than others from the same group (i.e., based on same salt but different HBD). In addition, the presence of ChCl salt in the DES increased the weight loss of the treated samples to a greater extent than samples treated with DESs based on other salts. Therefore, ChCl-based DESs are considerably more effective in providing a higher level of functionalization than N,N- and MPB-based DESs.

The final estimate concerning the role of DESs in eliminating and/or adding new functional groups was determined through the analysis of data from Raman, XRD, and FTIR spectroscopy as well as thermogravimetric results, which are discussed in the following sections.

### Raman spectroscopy

Raman spectroscopy was performed to study the changes in the structure of the graphene after each treatment. The Raman spectra for each group of graphene samples were studied individually and compared with p-Gr and o-Gr. An overall comparison is provided in Additional file 5: Table S21 in the Supplementary. As was the case for the Raman spectra of graphene-based materials, the prominent peaks at approximately 2700 cm^-1^, 1580 cm^-1^, and 1340 cm^-1^ refer to G′, G, and D bands, respectively. The most intense peak among all of the spectra was found for the G band, a result that was similar to that of a previous study that used the same AO-4 grade graphene nanopowder [52]. Generally, the G band is related to sp^2^-bonded carbon atoms in the hexagonal lattice of the graphitic structure. However, the D band reflects the presence of defects or disorders caused by sp^3^ hybridized carbon in the lattice, while G′ is caused by second-order, zone-boundary phonons that are related to the assembly of the multilayers [8, 53]. The intensity ratio of I_D_/I_G_ provides an indication of degree of oxidation and/or covalent functionalization [7]. The appearance of the G′ band (i.e., sharp or overlapped peak), intensity (relative to other bands), and frequency shifts also are important in predicting the number of layers in multi-layer graphene [54, 55].

**Fig. 8:**
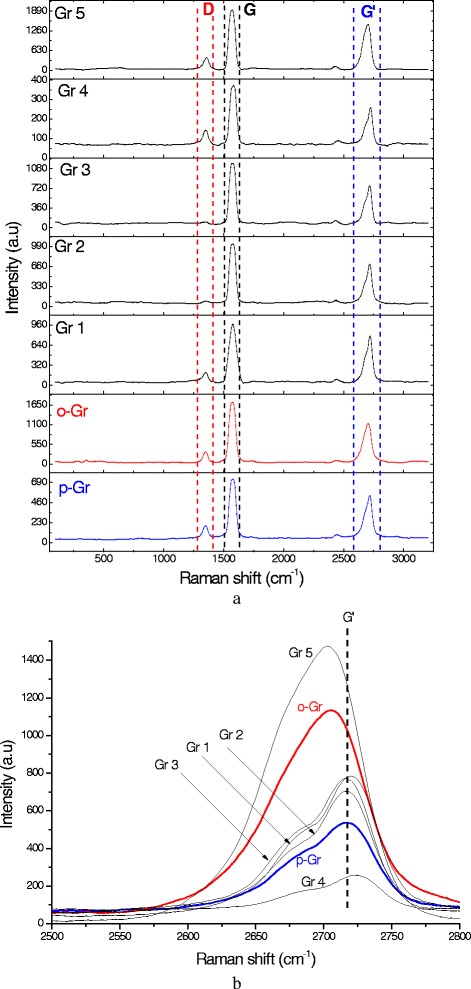
**a** Raman spectra of Gr 1–5 in comparison to pristine and oxidized graphene (p-Gr and o-Gr, blue and red respectively), **b** comparison of G′ bands

In previous studies [8, 56, 57], the researchers stated criteria to predict the number of graphene layers based on the G′ band. By applying the same criteria on the Raman spectrum of p-Gr in the present work (Fig. 8), the number of layers was predicted to be greater than 10. As expected, graphene oxidation led to a higher I_D_/I_G_ value due to the deformation caused by the introduction of oxygen-containing functional groups. This deformation was not as significant as it was for common o-Gr produced by Hummers’ method starting from graphite. The reason was the use of a non-destructive ultrasonic method in the pretreatment and the low deformation level of p-Gr. The G′ band shifted to a lower frequency (Additional file 5: Table S21) and became sharper and more intense. These observations are strong evidence of a successful oxidation process that resulted in the reduction of the particle size of the graphene and decreased the number of layers by the exfoliation effect.

Additional file 1: Figures S57-S59 in the Supplementary Appendix show the rest of the Raman spectra for Gr 6–18 arranged in groups as well as the difference in the G′ band. After treatment with DES, a high level of exfoliation was still expected in Gr 5, 7, 10, 16, 17, and 18 due to the sharp G′ band compared to the band in p-Gr and the shift to lower frequencies.

Gr 18 was found to have the highest deformation level, which can be attributed to the effect of its high acidity based on ChCl:MA. However, considerable increases in I_D_/I_G_ also were recorded in Gr 4, 15, 13, 8, and 10 as a result of covalent functionalization. By contrast, Gr 2, 3, 6, 7, 9, 12, 14, and 17 seemed to restore their graphitic structure by healing the defects caused after oxidation. This case might represent a clear deoxidation effect, which was confirmed by TGA and XRD. Slight increases in I_D_/I_G_ values were reported for the other samples (i.e., Gr 1, 5, 11, and 16), reflecting the simultaneous effects of functionalization and deoxidation.

Although an increased I_D_/I_G_ ratio is expected after oxidation, some researchers have reported that the reduction effect resulted in a lower ratio [58–60]. Nevertheless, it should be stated that multi-layer restacking, exfoliation, and deformation also have effects on the ratio. Exfoliation can cause a significant increase in defected sites, thus a higher I_D_/I_G_ is expected. Conversely, the restacking of the graphene layers can result in a decrease in the ratio [11]. Reduction was almost always accompanied by an increased I_D_/I_G_, especially after the chemical reduction of o-Gr produced by Hummers’ method. However, this is in disagreement with other studies [61, 62].

In the case of oxidation, the I_D_/I_G_ ratio is increased. In contrast, this ratio could be either increased or decreased with reduction. Two possible reduction pathways could be proposed, i.e., 1) the decrease in I_D_/I_G_ indicates the healing effects that are caused when the sp^2^ double bonds are restored in graphene and 2) constant or increased I_D_/I_G_ values indicate that the sp^3^ structure has been preserved, which could the result of the addition of different functional groups accompanied by the reduction reaction.

**Fig. 9:**
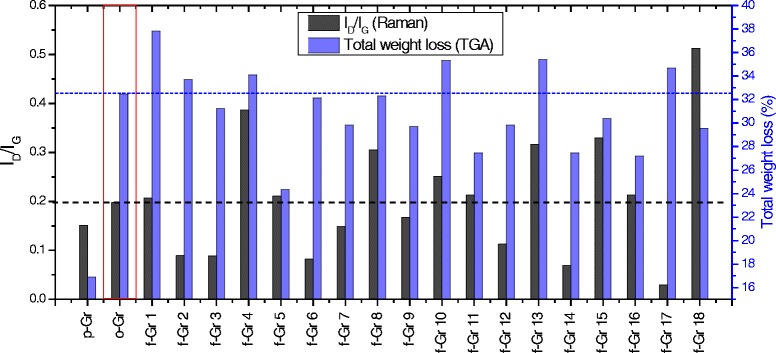
Combination data of I_D_/I_G_ and total weight loss in graphene samples as extracted from Raman and thermogravimetry studies

The reduction effect in this study was confirmed by the TGA and XRD results. It was assumed that a greater reduction level would be accompanied by less weight loss (from TGA) than occurred for o-Gr. However, reduction also can occur in parallel with functionalization, i.e., replacing oxygen groups with other groups from DES. Figure 9 summarizes the information from the Raman spectra and the TGA curves for all of the treated graphene samples. The figure shows that Gr 5 had the minimum weight loss during the TGA test among the DES-treated samples. This was supported by a slight increase in I_D_/I_G_. Thus, ChCl:U was expected to produce a deoxidation effect as a result of the replacement of some of the oxygen-containing functional groups with other groups from DES (as detected by FTIR spectroscopy). The alkalinity of this type of DES could be responsible for this effect, which would be in agreement with previous findings that alkaline solutions are good deoxygenating agents for o-Gr [43]. The deoxidation effect apparently was achieved for most of the DES-functionalized graphene, but it occurred at different levels. Compared to o-Gr, higher values of total weight loss and I_D_/I_G_ ratio were reported for Gr 1, 4, 10, and 13, reflecting a lower level of reduction than occurred in the other samples. It was anticipated that Gr 18 would undergo elimination of the functional group and deformation of its structure because it presented the highest I_D_/I_G_ with a lower weight loss than o-Gr.

### XRD analysis

Additional file 1: Figure S60 in the Supplementary Appendix shows the XRD patterns of p-Gr, o-Gr, and DES-functionalized graphene oxide (Gr 1–18). The peaks observed at 2θ = 26.7, 44.7, 54.7, and 77.7° in p-Gr represent the crystalline planes (002), (101), (004), and (110). This agreed with the findings of a previous study that used the same type of graphene [63]. After mild oxidation with KMnO_4_, a prominent diffraction peak appeared at 2θ = 11.16°, reflecting the interlayer spacing of 0.79 nm (according to Bragg’s law [64, 65]). The small peak at 2θ = ~26° suggests the existence of unoxidized graphene [66]. In such cases, the actual crystallinity of the graphene can be restored only after a reduction reaction [53].

Table 9 lists information related to the peaks of the (002) and (004) graphitic planes, as extracted from the XRD patterns, for all graphene samples that were studied. After the DES treatment, the exfoliation or restacking level will be greater than that of p-Gr, and it can be followed easily from the shifting in diffraction peaks (to lower or higher 2θ degrees). The emergence/disappearance of peaks and the decrease in their intensities also can be interpreted by DES functionalization role. The breadth of the (002) peak increases with the increasing number of graphitic layers per each crystalline [67].

**Table 9:** Diffraction peak information from XRD patterns of p-Gr and DES-treated graphene oxide

Sample	Diffraction peak of (002)		Diffraction peak of (004)	
	2θ (deg)	Intensity	2θ (deg)	Intensity
p-Gr	26.70	1682.26	54.74	97.48
Gr 1	26.65	693.40	54.72	29.00
Gr 2	26.76	859.81	54.96	55.24
Gr 3	26.55	387.27	54.70	24.99
Gr 4	26.55	387.27	54.65	30.86
Gr 5	26.53	137.47	54.68	13.03
Gr 6	26.53	216.22	54.70	13.00
Gr 7	26.72	588.45	54.86	22.99
Gr 8	26.76	937.83	54.84	43.40
Gr 9	26.74	690.84	55.00	36.15
Gr 10	26.62	257.12	54.68	14.00
Gr 11	26.84	930.79	55.04	39.39
Gr 12	26.86	525.88	54.94	37.09
Gr 13	26.87	670.58	55.04	35.05
Gr 14	26.78	375.97	54.90	22.11
Gr 15	26.94	842.81	55.04	32.99
Gr 16	26.76	672.86	54.84	45.41
Gr 17	26.72	644.28	54.82	39.03
Gr 18	26.94	1302.70	55.06	54.99

As shown in Additional file 1: Figure S60, all of the XRD patterns had new peaks at ~26.6°, which corresponds to the typical graphene diffraction peak [68]. This confirmed that a partial reduction reaction occurred after the DES treatment.

For Gr 1 modified by ChCl:Gly, the first peak in the XRD pattern appeared at 2θ = 18.22°, reflecting a preserved form of graphene oxide that was partially reduced by the DES. The prominent peak at 2θ = 26.64° was observed as an indication of the reduced form of graphene.

**Fig. 10:**
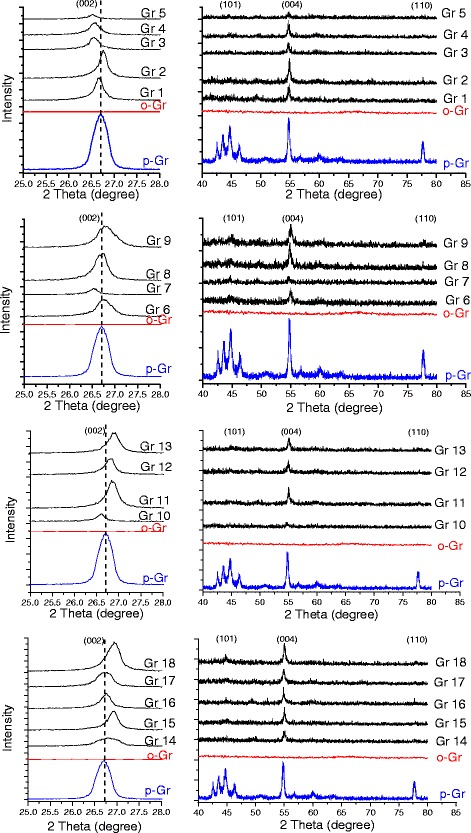
Comparison between diffraction peaks from XRD patterns of different treated and untreated graphene

The intensities of the peaks of all DES-treated samples were lower than those in p-Gr. This behavior frequently has been reported to be the result of functionalization, especially when it is accompanied by peak shifting to less than 2θ degrees [69–71]. Figure 10 shows the XRD diffraction peaks in the different functionalized groups compared to p-Gr and o-Gr. The peaks were compared using suitable offset values and by maintaining a constant relative intensity difference. By comparing the graphene groups, it can be concluded that treating graphene with ChCl-based DES (Group 1) leads to a graphene structure with a lower stacking form than the other groups. The diffraction peaks in this group were observed at lower 2θ values than p-Gr and the other treated samples. This indicated that there was a larger interlayered spacing between the sheets caused by the added functional groups from the DES source. However, in Group 2, the positions of the peaks were almost identical to those in p-Gr (expect for Gr 7), and the structural characteristics were restored due to the reduction effect. However, treating with MPB-based DES (Group 3) resulted in lower interlayered spacing than existed in the original p-Gr (higher 2θ values). Only Gr 10 did not experience this effect, and a slight decrease in 2θ was recorded. The same effect also was observed in Group 4, which presented a higher stacking level than p-Gr.

It can be expected that the presence of new functional groups from the DES sources contributed to changing the structure such that it differs from that of p-Gr. For example, if the reduction reaction took place and new hydrophobic functional groups were added, the π-π stacking between the layers would be enhanced to a higher level than the stacking in p-Gr. In contrast, the addition of hydrophilic functional groups (which replace the existing oxygen groups) would result in a lower stacking level than that of a fully-cleaned surface. To prove this, dispersibility tests were conducted in aqueous solutions. The improvement or deterioration in graphene dispersibility compared to o-Gr and p-Gr confirmed the roles of the hydrophilic or hydrophobic functional groups that possibly were attached after treatment with DES.

### SEM, TEM, and particle size analyses

**Fig. 11:**
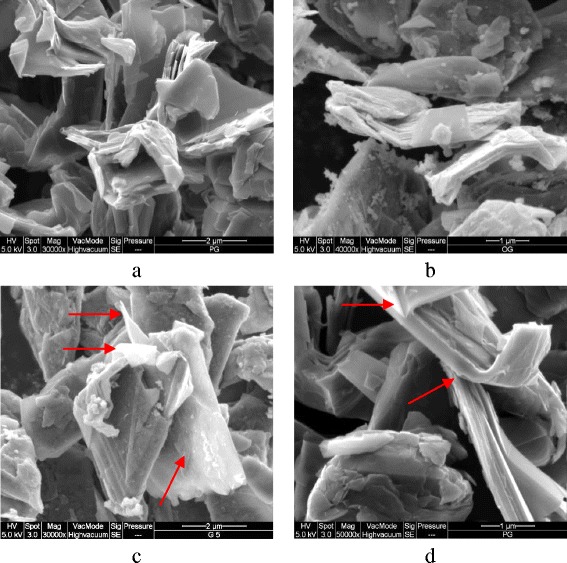
FE-SEM images of pristine graphene, oxidized and DES 5-modified and DES 18-modified graphene (**a**, **b**, **c** and **d** respectively)

**Fig. 12:**
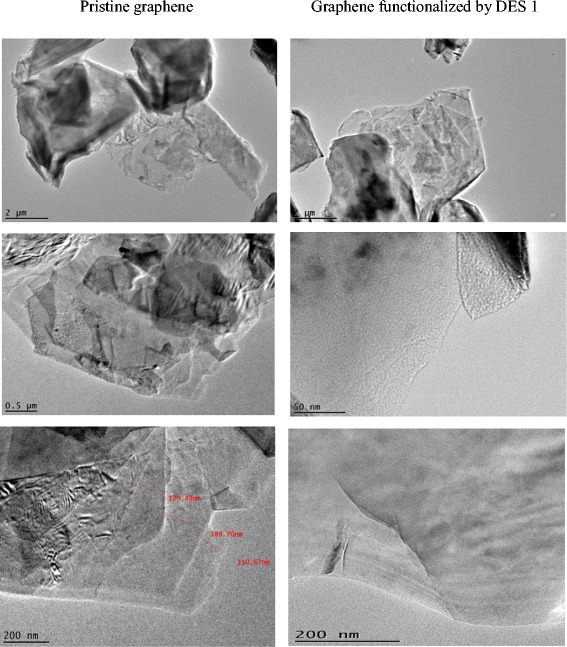
TEM images for pristine and functionalized graphene by DES 1

A few samples were selected at random for the tests to provide a general vision of the impact of DESs on the structure of graphene. Figure 11 shows FE-SEM images of (a) untreated graphene, (b) oxidized graphene, and graphene modified by (c) DES 5 and (d) DES 18. In Fig. 11(b), the white spots on the oxidized surface of the graphene represent the residual oxidants that can be targeted usefully by DES molecules. Due to the completely dry form of the samples, no considerable difference was evident in the structure of the graphene before and after treatment. However, in DES 5-functionalzed graphene (Fig. 11c), some loose stacking between the outer layers and other layers were observed. This loose arrangement of sheets is a sort of deformation of the structure that can improve the dispersibility of graphene in a water medium. Furthermore, traces of deformation obviously were found in the graphene sheets modified by DES 18 (Fig. 11(d)). Therefore, DES-based treatment can be useful for the preparation of a higher level of functionalization. TEM images of pristine and functionalized graphene proved to have a higher exfoliation level of DES 1-functionalized graphene than p-Gr. A wider area of transparent graphene layers were observed after functionalization, which is a reflection of a monolayer or a double layer of graphene, while most areas of untreated graphene were found to be darker, corresponding to the presence of multi-layer sheets [72]. Figure 12 shows a clear single layer in the treated sample, whereas a single graphene layer rarely is detected in pristine graphene.

The particle sizes of graphene modified by DES 5 and 18 were investigated similarly in p-Gr and o-Gr (Additional file 1: Figure S61). The results showed a decrease of ~50% in their average size compared to o-Gr particles. This also confirmed that the deformation of the structure was caused by serious splitting of the aggregated particles. The test may not exhibit the real values for the sizes of the graphene particles, but it does provide an indication concerning the overall changes in particle size after treatment with DES.

### Dispersibility and suspension stability

**Fig. 13:**
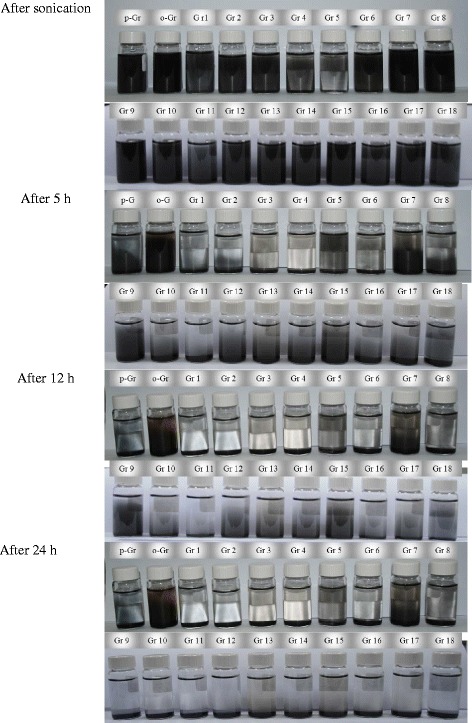
Suspension behavior of pristine graphene (p-Gr), oxidized graphene (o-Gr) and the ones treated with DES 1 to DES 18 (Gr 1 to Gr 18) in distillated water during the first 24 h

Tests of the dispersibility and suspension stability were conducted for the graphene samples using the same conditions of concentration, sonication time, and temperature (room temperature). The first dispersibility test included the preparation of graphene suspensions with relatively high concentrations, i.e., 1 mg/ml, and the test continued for 24 h. These conditions were used to accelerate the precipitation, thereby expediting the comparisons of the treated and untreated samples. Figure 13 shows that all of the graphene samples were dispersed homogeneously in water for the first 5 h, except for the samples that were functionalized with DES 3, 4, 6, and 11 (Gr 3, Gr 4, Gr 6, and Gr 11). This was because the graphene agglomeration in these four sets of samples was enhanced after treatment with DES. This confirmed the partial elimination of oxygen-containing functional groups that had been identified previously for these samples. Therefore, the hydrophilic effect of the remaining groups was at a lower level, thus causing insufficient dispersibility. After 12 h, graphene samples functionalized by DES 1, 2, 3, 4, 6, 8, 11, 14, and 16 showed a short-term stability with a clear liquid appearance and a precipitated graphene layer at the bottom. The other samples exhibited longer stability, with the best results reported for graphene functionalized with DES 5, 7, and 9 (Gr 5, 7, 9) and for oxidized graphene (o-Gr). However, nearly all samples were fully precipitated after a settling time of 24 h. Generally, the dispersibility of the functionalized graphene oxide samples was determined by using the level of deoxidation achieved, the nature of the new functional groups, and the average size of the resulting particles.

Note that many parameters affect the duration of a stable dispersion. In a previous study on the aqueous dispersion of graphene, Ding et al. [73] found that sonication time, temperature, and the concentration of graphene determined the long-term stability. Stability was achieved for a period ranging from two weeks to two months by adjusting the sonication time and temperature within the ranges 2–6 h and 160–220 °C, respectively, at concentrations below 1 mg/ml.

**Fig. 14:**
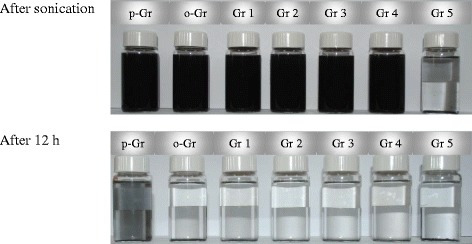
Suspension behavior of pristine graphene (p-Gr), oxidized graphene (o-Gr) and the ones treated by DES 1 to DES 5 (Gr 1 to Gr 5) in acetone during 12 h

**Fig. 15:**
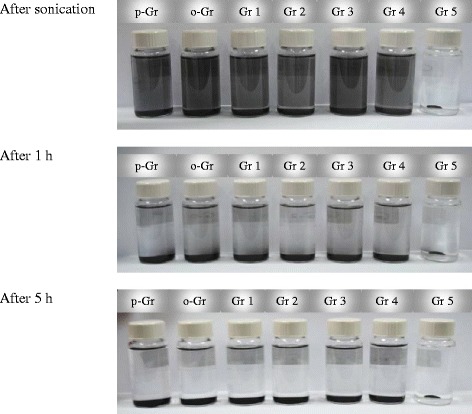
Suspension behavior of pristine graphene (p-Gr), oxidized graphene (o-Gr) and the ones treated by DES 1 to DES 5 (Gr 1 to Gr 5) in n-hexane during 5 h

Furthermore, dispersibility in acetone was monitored for the five types of modified graphene, i.e., Gr 1–5 (Fig. 14). Dispersions were prepared at the same concentration of graphene that was used earlier in aqueous suspensions. After only 12 h, the graphene powder precipitated as a result of the chemical modification. The oxidized and functionalized graphenes (o-Gr and Gr 1 to 5) have less dispersion stability than the untreated graphene (p-Gr). Gr 5 could not be dispersed in acetone, even after sonication and hard shaking. This can be attributed to the isolation role resulting from the functional groups from ChCl:U being attached to the surface of the graphene because these groups are immiscible in acetone. For the same graphene group, we also assessed the dispersibility in n-hexane as a non-polar solvent. Figure 15 shows that the stability was short-term and that full precipitation occurred after only 5 h. Sample Gr 5 could not be dispersed even after 3 h of sonication. The graphene particles formed large, immiscible aggregates that precipitated at the bottom of the vial for the entire duration. This also confirmed the successful functionalization of graphene oxide. The nature of the newly-added functional groups was identical to the nature of the DES molecules. In agreement with the previous findings (FTIR and Raman spectroscopy), these functional groups contained oxygen and nitrogen, and they were chemically attached to the graphene. The high hydrophilicity of these groups prevented the nanosheets from scattering throughout the solution. Thus, they showed no affinity to be dissolved in n-hexane, which was the same as the nature of the original ChCl:U DES.

Dispersibility tests in different polar and non-polar solvents can be used to determine the nature of the surface and the exact hydrophilicity and hydrophobicity levels of the graphene nanosheets. The stability of the suspension in a non-polar solvent, i.e., n-hexane, was very short for the samples that were tested before and after treatment with DES. Conversely, stability in water was better for all samples, and it lasted for a longer period of time in some cases. Zhang *et al.* reported similar stability results in several polar solvents [74]. By treating oxidized graphene with IL 1-allyl-3-methylimidazolium chloride, they produced a water-soluble graphene. A dispersibility test showed that such water-soluble graphene would remain dispersed for more than 36 h at a concentration of 1 mg/ml. By contrast, when the solvents dimethyl sulfoxide and 2-propanol were used instead of water, a black precipitate was found at the bottom of the vials, reflecting lower stability levels.

**Fig. 16:**
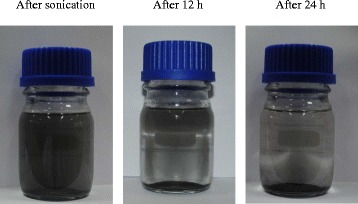
Suspension behavior for a mixture of poorly dispersed graphene samples (1, 2, 3, 4, 6, 8, 11, 14 and 16) in distillated water in 24 h

In a further investigation, a suspension was made from poorly dispersed graphene in distilled water (1, 2, 3, 4, 6, 8, 11, 14, and 16). The resulting mixtures were stable for longer periods of time than other suspensions, exceeding 24 h, as shown in Fig. 16. This reflects the complicated interactions between different functionalized graphene sheets.

Solvation in water generally is caused by the hydrogen bonds between the graphene sheets and the water molecules [75], but when the van der Waals interaction is stronger between the sheets, their exfoliation is prevented. Oxygen-containing functional groups help to alter the van der Waals interactions between sheets and render them hydrophilic [76].

As mentioned before, the aim of this section was to show the differences in the behaviors of the dispersions among all of the graphene samples. The stability of their dispersion can be prolonged if various conditions, such as sonication time, concentration, and temperature, are adjusted properly to optimum levels. Choosing a suitable type of dispersant with or without the addition of dispersing aids also is important when stability over time is required [77].

**Fig. 17:**
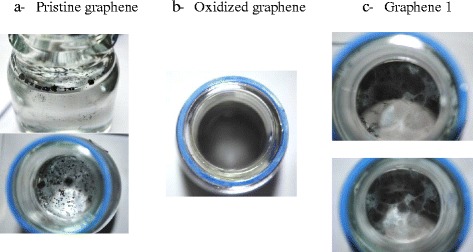
First action of different graphene samples towards water addition, pristine (**a**), oxidized (**b**) and the modified with DES 1 (**c**)

Changes in the surfaces of the graphene after treatment with DES sometimes resulted in greater decrease in the stability of the dispersions of DES-functionalized graphene than oxidized graphene due to the reduction effect. To explain this behavior, a small experiment was conducted to determine how graphene particles reacted when immersed in an aqueous solution. The first moments after water was added to the graphene samples were monitored for p-Gr, o-Gr, and Gr 1 (Fig. 17). Fast agglomeration of the pristine carbon particles occurred when the water was added (Fig. 17(a)). The hydrophobic property and surface chemistry of pristine graphene accounted for this behavior [78, 79]. By contrast, Fig. 17(b) shows that the presence of new hydrophilic groups after treatment with KMnO_4_ led to instantaneous homogenization immediately after water was added to o-Gr. This phenomenon was similar to mixing water with a soluble black pigment powder, and it resulted from the higher surface energy of o-Gr [77].

However, Fig. 17(c) shows that the graphene nanosheets that were functionalized by DES 1 based on ChCl:Gly tended to form a transparent, silver-colored layer floating on the water/air interface. Along with the deoxidation effect, as discussed earlier, this may indicate a double-sided functionalization of the graphene bilayer, followed by the exfoliation process between the nanosheets [49]. Eventually, this resulted in uneven levels of functionalization between the exfoliated layers. Thus, just after sonication, the layers aligned into dendritic architectures via hydrogen bonding and the ion-dipolar interactions between the carboxyl groups and water molecules [80].

#### Zeta potential

**Fig. 18:**
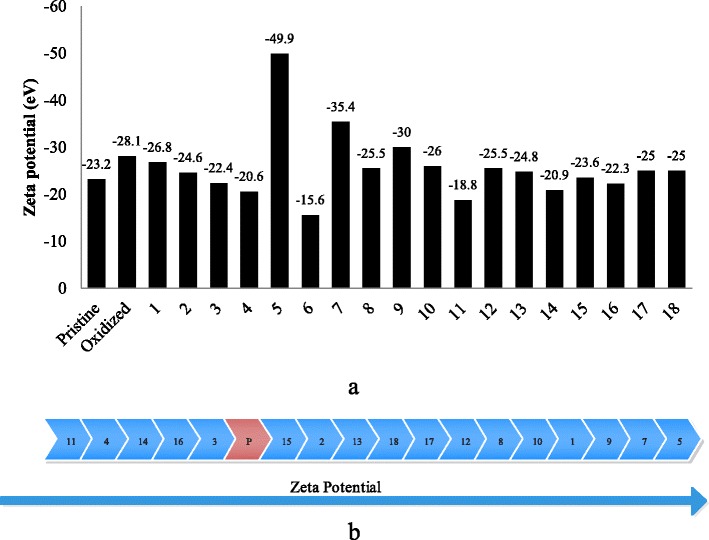
**a** Zeta potential results for pristine and functionalized graphene suspensions in water (0.01g/100ml), **b** Arrangements of Zeta Potential values of all tested graphene accordingly

Zeta potential was measured for graphene dispersions in water (0.01 g/100 ml), and the results are presented in Fig. 18(a). The maximum enhancement in zeta potential after treatment with KMnO_4_ was for the graphene that was functionalized by DES 5 based on ChCl:U. However, not all graphene functionalization processes led to better dispersibility. This can be attributed to changes on the surface of the carbon after each treatment due to the manipulation of functional groups that have different hydrophilicity and hydrophobicity properties. It also confirmed the reduction effect caused by the DESs, which control the presence level of oxygen-containing functional groups in the modified samples. These groups led to electronegativity with the different values of electron density responsible for the negative values of zeta potential. However, according to Riddick [81], a zeta potential value greater than 30 eV suggests that the solution will be stable for a reasonable amount of time, and, if the value exceeds 40 eV, the solution can be considered to be much more stable. Accordingly, Gr 9 and 7 are stable in a water medium, but they are not as stable as a well-dispersed functionalized graphene by DES 5. The oxidation process enhanced the stability by 21%, because it helped to increase the hydrophilicity and the extent of exfoliation.

Dispersibility improvements in Gr 5, 7, and 9 can be ascribed to the simultaneous functionalization and reduction effects resulting from the respective DESs. In some cases, the zeta potential values decreased after the DES treatment. However, the overall difference between pristine and DES-modified graphene indicated substantial improvements, especially for Gr 5, 7, and 9. Treating with DES 3, 4, 6, 11, 14, and 16 changed the surface chemistry such that their dispersion stability in water was diminished. Based on FTIR analyses of these samples, −C-H and -CH_2_ bonds commonly are present on the surface of the carbon in samples that have zeta potential values less than 20 eV. Hydrophobic affinity for these functional groups was anticipated to influence the solubility of graphene in water. In general, the deterioration of dispersibility can be explained by (a) the effect of the removal of some hydrophilic functional groups from the surface of the carbon caused by the DESs, e.g., the deoxidization effect mentioned earlier in Section 3.5; (b) the addition of new functional groups that could have an adverse effect on the stability of the dispersion in water; and (c) the physical adsorption of DES materials occurring on active sites on the surface of the carbon through irreversible, non-covalent modification [43], which has been reported in most studies to be the result of π–π stacking between the graphene plane and aromatic molecules when they are present [82]. Figure 18(b) shows the arrangement of the graphene samples, from the lowest to the highest, according to the values of their zeta potentials.

To investigate the role of DES and the prior treatment with KMnO_4_ further, pristine graphene flakes were treated directly with some types of DES without prior purification. For the flakes treated with DES 1 and DES 5, there were no changes in the values of the zeta potentials. This is also in accordance with the FTIR results, which confirmed the need for pretreatment with KMnO_4_.

Modified graphene can be produced and used in various functional applications. Graphene with more hydrophobic affinity can be used as additives in polymer-based composites [83]. In addition, all of the functionalized samples may be used in selective sorption applications, especially of positively-charged metallic ions.

## Conclusions

The use of DESs as functionalizing agents for graphene-based materials was investigated on graphene oxide. Chemical changes on the surface of the graphene were studied using FTIR spectra. The thermal stability obtained from simultaneous TGA/DTG analyses showed various stability changes when compared with pristine and oxidized graphene. Raman spectroscopy and XRD results also identified chemical and structural changes. Different levels of reduction were achieved after DES treatment, and simultaneous functionalization/reduction was reported in some cases. Further investigations of the structural changes were performed using SEM, TEM, and particle size analyses. The dispersibility of the treated graphene also was assessed and characterized based on zeta potential values. The results provided convincing indication of the changes on surface chemistry that occurred after DES treatment. However, considerable improvements in the dispersion stability of the graphene/water systems were recorded for graphene treated with DES 5, 7, and 9 compared to graphene oxide. The introduction of new hydrophilic functional groups was responsible for this behavior. The different reducing abilities of the DESs used were responsible for the different levels of agglomeration during the dispersibility tests. However, each modification process was an independent case that could be interpreted based on various aspects. The as-functionalized graphene may have different electrical, mechanical, or optical properties than the original pristine graphene. Thus, it is important to extend this study and test the functionalized graphene in various potential applications.

## Additional files

Additional file 1: Figures S1-S61. FTIR spectra of DESs and their individual components as well as DES-modified graphene oxide, TGA/DTG curves for DES-functionalized graphene oxide (Gr 1–18), Raman Spectra (for Gr 6–18), XRD patterns (Gr 1–18) and particle size distribution (Gr 5 and Gr 18). Additional file 2: Tables S1-S18. Detected peaks form FTIR spectra for DESs and their individual components. Additional file 3: Table S19. Detected FTIR peaks from DES spectra with the matching functional groups and origin. Additional file 4: Table S20. Comparison between FTIR spectra of all graphene samples showing the appearance/disappearance of peaks after each treatment. Additional file 5: Table S21. Description of data: Extracted information from Raman spectra of pristine, oxidized and DES-functionalized graphene oxide.
