# Supplementary material for: Functionalization of graphene using deep eutectic solvents
Source: Nanoscale Res Lett. 2015 Aug 12;10:324. doi: 10.1186/s11671-015-1004-2 (PMC4531886; doi:10.1186/s11671-015-1004-2)
Supplement: Additional file 1: Figures S1-S61. — FTIR spectra of DESs and their individual components as well as DES-modified graphene oxide, TGA/DTG curves for DES-functionalized graphene oxide (Gr 1–18), Raman Spectra (for Gr 6–18), XRD patterns (Gr 1–18) and particle size distribution (Gr 5 and Gr 18). [file 11671_2015_1004_MOESM1_ESM.docx]

For Figures S. 1 to 18

W (if existed)

DES

Salt

HBD

Figure S 1. FTIR spectra for DES 1, ChCl and Gly

Figure S 2. FTIR spectra for DES 2, ChCl and EG

Figure S 3. FTIR spectra for DES 3, ChCl and DEG

Figure S 4. FTIR spectra for DES 4, ChCl and TEG

Figure S 5. FTIR spectra for DES 5, ChCl and U

Figure S 6. FTIR spectra for DES 6, N,N and Gly

Figure S 7. FTIR spectra for DES 7, N,N and EG

Figure S 8. FTIR spectra for DES 8, N,N and DEG

Figure S 9. FTIR spectra for DES 9, N,N and TEG

Figure S 10. FTIR spectra for DES 10, MPB and Gly

Figure S 11. FTIR spectra for DES 11, MPB and EG

Figure S 12. FTIR spectra for DES 12, MPB and DEG

Figure S 13. FTIR spectra for DES 13, MPB and TEG

Figure S 14. FTIR spectra for DES 14, ChCl, Glu and W

Figure S 15. FTIR spectra for DES 15, ChCl, Fru and W

Figure S 16. FTIR spectra for DES 16, ChCl, Suc and W

Figure S 17. FTIR spectra for DES 17, ChCl, Gly and W

Figure S 18. FTIR spectra for DES 18, ChCl and MA

Figure S 19. FTIR spectrum for pristine graphene

Figure S 20. FTIR spectrum for oxidized graphene

Figure S 21. FTIR spectrum for DES 1-modified graphene

Figure S 22. FTIR spectrum for DES 2-modified graphene

Figure S 23. FTIR spectrum for DES 3-modified graphene

Figure S 24. FTIR spectrum for DES 4-modified graphene

Figure S 25. FTIR spectrum for DES 5-modified graphene

Figure S 26. FTIR spectrum for DES 6-modified graphene

Figure S 27. FTIR spectrum for DES 7-modified graphene

Figure S 28. FTIR spectrum for DES 8-modified graphene

Figure S 29. FTIR spectrum for DES 9-modified graphene

Figure S 30. FTIR spectrum for DES 10-modified graphene

Figure S 31. FTIR spectrum for DES 11-modified graphene

Figure S 32. FTIR spectrum for DES 12-modified graphene

Figure S 33. FTIR spectrum for DES 13-modified graphene

Figure S 34. FTIR spectrum for DES 14-modified graphene

Figure S 35. FTIR spectrum for DES 15-modified graphene

Figure S 36. FTIR spectrum for DES 16-modified graphene

Figure S 37. FTIR spectrum for DES 17-modified graphene

Figure S 38. FTIR spectrum for DES 18-modified graphene

Figure S 39 TGA and DTG thermograms of DES 1-functionalized graphene (Gr 1)

Figure S 40 TGA and DTG thermograms of DES 2-functionalized graphene (Gr 2)

Figure S 41 TGA and DTG thermograms of DES 3-functionalized graphene (Gr 3)

Figure S 42 TGA and DTG thermograms of DES 4-functionalized graphene (Gr 4)

Figure S 43 TGA and DTG thermograms of DES 5-functionalized graphene (Gr 5)

Figure S 44 TGA and DTG thermograms of DES 6-functionalized graphene (Gr 6)

Figure S 45 TGA and DTG thermograms of DES 7-functionalized graphene (Gr 7)

Figure S 46 TGA and DTG thermograms of DES 8-functionalized graphene (Gr 8)

Figure S 47 TGA and DTG thermograms of DES 9-functionalized graphene (Gr 9)

Figure S 48 TGA and DTG thermograms of DES 10-functionalized graphene (Gr 10)

Figure S 49 TGA and DTG thermograms of DES 11-functionalized graphene (Gr 11)

Figure S 50 TGA and DTG thermograms of DES 12-functionalized graphene (Gr 12)

Figure S 51 TGA and DTG thermograms of DES 13-functionalized graphene (Gr 13)

Figure S 52 TGA and DTG thermograms of DES 14-functionalized graphene (Gr 14)

Figure S 53 TGA and DTG thermograms of DES 15-functionalized graphene (Gr 15)

Figure S 54 TGA and DTG thermograms of DES 16-functionalized graphene (Gr 16)

Figure S 55 TGA and DTG thermograms of DES 17-functionalized graphene (Gr 17)

Figure S 56 TGA and DTG thermograms of DES 18-functionalized graphene (Gr 18)

|  |
| --- |
| a |
|   b |
| Figure S 57(a) Raman spectra of Gr 6-9 in comparison to pristine and oxidized graphene (p-Gr and o-Gr, blue and red respectively), (b) comparison of G′ bands. |

|  |
| --- |
| a |
| ****  b |
| Figure S 58 (a) Raman spectra of Gr 10-13 in comparison to pristine and oxidized graphene (p-Gr and o-Gr, blue and red respectively), (b) comparison of G′ bands. |
|  |
| a |
|   b |
| Figure S 59 (a) Raman spectra of Gr 14-18 in comparison to pristine and oxidized graphene (p-Gr and o-Gr, blue and red respectively), (b) comparison of G′ bands. |

|  |  |
| --- | --- |
| a | b |
|  |  |
| c | d |

Figure S 60XRD patterns of p-Gr, o-Gr and DES-functionalized graphene oxide arranged in groups 1 to 4 (a to d)

| 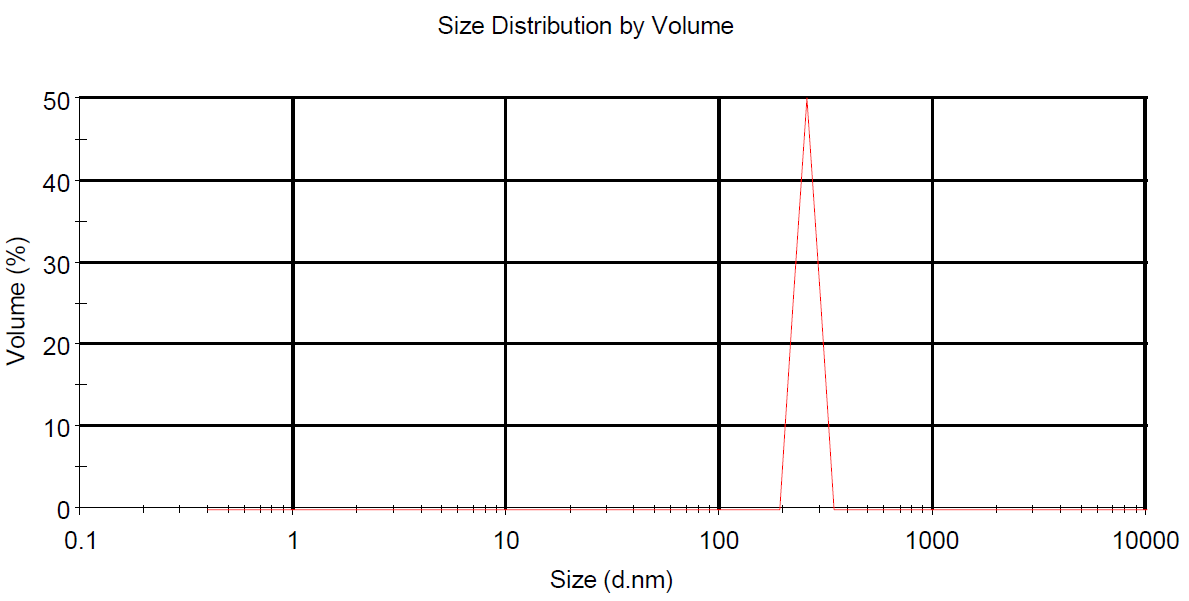 |
| --- |
| a |
| 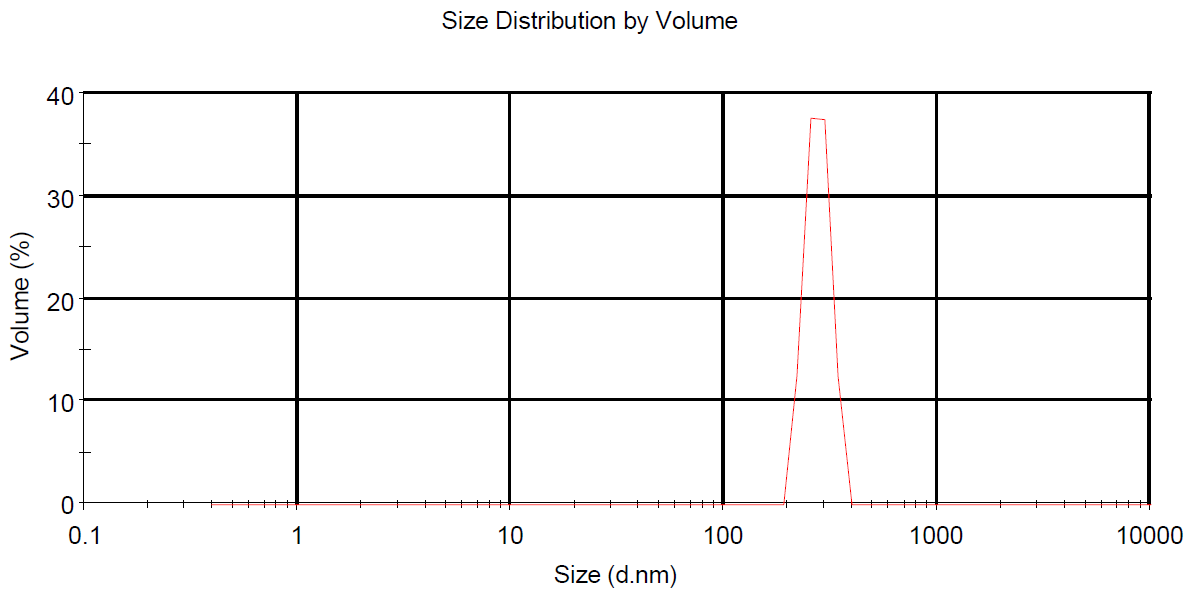 |
| b |

Figure S 61Size distribution of DES 5-functionalized graphene (a), Z-Average 0.2564 µM and DES 18-functionalized graphene (b), Z-Average 0.2766 µM.
