# Supplementary material for: Functionalization of graphene using deep eutectic solvents
Source: Nanoscale Res Lett. 2015 Aug 12;10:324. doi: 10.1186/s11671-015-1004-2 (PMC4531886; doi:10.1186/s11671-015-1004-2)
Supplement: Additional file 3: Table S19. — Detected FTIR peaks from DES spectra with the matching functional groups and origin. [file 11671_2015_1004_MOESM3_ESM.docx]

Table S. 19 Detected FTIR peaks from DES spectra with the matching functional groups and origin.

| DES | Wavenumber at peak (cm^-1^) | Functional Group | Origin |
| --- | --- | --- | --- |
| 1 | 3300 | O-H (stretching) | Gly |
|  | 2928, 2878 | sp^3^ hybridized C–H (stretching) | Gly |
|  | 1478 | CH_2_ (bending) | ChCl |
|  | 1109 | C–O (stretching) in 1° alcohol | Gly |
|  | 1036 | C-C-O (asymmetric stretching) | Gly |
|  | 864 | C-C-O (symmetric stretching) | Gly |
|  | 551 | O-H (Out-of-plane bending) | Gly |
| 2 | 3298 | O-H (stretching) | EG |
|  | 3028 | N-H (stretching) | ChCl |
|  | 2938, 2872 | sp^3^ hybridized C–H (stretching) | EG |
|  | 1479 | CH_2_ (bending) | ChCl |
|  | 1203 | C–O–H (bending) | EG |
|  | 1084 | C–O (stretching) in 1° alcohol | EG |
|  | 1036 | C-C-O (asymmetric stretching) | EG |
|  | 882 | C-C-O (symmetric stretching) | EG |
|  | 596 | O-H (Out-of-plane bending) | EG |
| 3 | 3299 | O-H (stretching) | DEG |
|  | 2922, 2870 | sp^3^ hybridized C–H (stretching) | DEG |
|  | 1478 | CH_2_ (bending) | ChCl |
|  | 1323 | C–O–H (bending) | DEG |
|  | 1325 | O-H (In-plane bending) | DEG |
|  | 1125 | C–O (stretching) in 1° alcohol | DEG |
|  | 1054 | C-C-O (asymmetric stretching) | DEG |
|  | 890 | C-C-O (symmetric stretching) | DEG |
|  | 867 | C–N^+^ (symmetric stretching) | ChCl |
|  | 608 | O-H (Out-of-plane bending) | DEG |
| 4 | 3309 | O-H (stretching) | TEG |
|  | 2868 | sp^3^ hybridized C–H (stretching) | TEG |
|  | 1477 | CH_2_ bond bending vibration | ChCl |
|  | 1166 | C–O bond stretching in 1° alcohol | TEG |
|  | 1060 | C-C-O (asymmetric stretching) | TEG |
|  | 885 | C-C-O (symmetric stretching) | TEG |
|  | 868 | C–N^+^ symmetric stretching vibration | ChCl |
|  | 574 | O-H (Out-of-plane bending) | TEG |
| 5 | 3314 | Symmetric NH_2_ stretch | U |
|  | 3186 | N-H (stretching) / O-H (stretching) | ChCl |
|  | 1660 | C=O (stretching) for amide | U |
|  | 1606 | N–H scissoring band | U |
|  | 1477 | CH_2_ (bending) | ChCl |
|  | 1434 | C–N (stretching) | U |
|  | 865 | C–N^+^ (symmetric stretching) | ChCl |
|  | 787 | N–H (out-of-plane bending band) | U |
| 6 | 3307 | O-H (stretching) | Gly |
|  | 2936, 2880 | sp^3^ hybridized C–H (stretching) | Gly |
|  | 1462 | CH_2_ (bending) | Gly |
|  | 1398 | C-H (deformation) | N,N |
|  | 1228 | C–O–H (bending) | Gly |
|  | 1109 | C–O (stretching) in 1° alcohol | Gly |
|  | 1030 | C-C-O (asymmetric stretching) | Gly |
|  | 855 | C-C-O (symmetric stretching) | Gly |
|  | 553 | O-H (Out-of-plane bending) | Gly |
| 7 | 3290 | O-H (stretching) | EG |
|  | 2936, 2880 | sp^3^ hybridized C–H (stretching) | EG |
|  | 2663, 2491 | N-H^+^ (stretching) of quaternary ammonium | N,N |
|  | 1460 | CH_2_ (bending) | EG |
|  | 1397 | C-H (deformation) | N,N |
|  | 1326, 1254, 1205 | C–O–H (bending) | EG |
|  | 1083 | C–O (stretching) in 1° alcohol | EG |
|  | 1032 | C-C-O (asymmetric stretching) | EG |
|  | 882 | C-C-O (symmetric stretching) | EG |
|  | 580 | O-H (Out-of-plane bending) / C–N^+^ (symmetric stretching) | EG / N,N |
| 8 | 3321 | O-H (stretching) | DEG |
|  | 2936, 2874 | sp^3^ hybridized C–H (stretching) | DEG |
|  | 1455 | CH_2_ (bending) | DEG |
|  | 1397 | C-H (deformation) | N,N |
|  | 1352, 1231 | C–O–H (bending) | DEG |
|  | 1125 | C–O (stretching) in 1° alcohol | DEG |
|  | 1051 | C-C-O (asymmetric stretching) | DEG |
|  | 892 | C-C-O (symmetric stretching) | DEG |
|  | 577 | O-H (Out-of-plane bending) / C–N^+^ (symmetric stretching) | N,N / DEG |
| 9 | 3331 | O-H (stretching) | TEG |
|  | 2868 | sp^3^ hybridized C–H (stretching) | TEG |
|  | 2491 | N-H^+^ (stretching) of quaternary ammonium | N,N |
|  | 1454 | CH_2_ (bending) | TEG |
|  | 1404 | C-H (deformation) | N,N |
|  | 1116 | C–O (stretching) in 1° alcohol | TEG |
|  | 1058 | C-C-O (asymmetric stretching) | TEG |
|  | 885 | C-C-O (symmetric stretching) | TEG |
|  | 563 | O-H (Out-of-plane bending) | TEG |
| 10 | 3297 | O-H (stretching) | Gly |
|  | 3029 | =C-H and ring C=C (stretching) | MPB |
|  | 2936, 2880 | sp^3^ hybridized C–H (stretching) | Gly |
|  | 1480 | =C-H and ring C=C (stretching) | MPB |
|  | 1417 | CH_2_ (bending) | Gly |
|  | 1210 | C–O–H (bending) | Gly |
|  | 1109, 1082, 952 | P-Phenyl (stretching) | MPB |
|  | 1109 | C–O (stretching) in 1° alcohol | Gly |
|  | 1039 | C-C-O (asymmetric stretching) | Gly |
|  | 864 | C-C-O (symmetric stretching) | Gly |
|  | 552 | O-H (Out-of-plane bending) | Gly |
| 11 | 3294 | O-H (stretching) | EG |
|  | 3021 | =C-H and ring C=C (stretching) | MPB |
|  | 2936, 2873 | sp^3^ hybridized C–H (stretching) | EG |
|  | 1484 | =C-H and ring C=C (stretching) | MPB |
|  | 1455 | CH_2_ (bending) | EG |
|  | 1326 | O-H (In-plane bending) / C–O–H (bending) | EG / MPB |
|  | 1135, 952 | P-Phenyl (stretching) | MPB |
|  | 1083 | C–O (stretching) in 1° alcohol | EG |
|  | 1038 | C-C-O (asymmetric stretching) | EG |
|  | 882 | C-C-O (symmetric stretching) | EG |
|  | 606 | O-H (Out-of-plane bending) | EG |
| 12 | 3290 | O-H (stretching) | DEG |
|  | 3023 | =C-H and ring C=C (stretching) | MPB |
|  | 2936, 2874 | sp^3^ hybridized C–H (stretching) | DEG |
|  | 1479 | =C-H and ring C=C (stretching) / CH_2_ (bending) | MPB / DEG |
|  | 1420 | P-CH_3_ (asymmetrically CH_3_ deformation) | MPB |
|  | 1326 | O-H (In-plane bending) / C–O–H (bending) | DEG |
|  | 1125 | C–O (stretching) in 1° alcohol | DEG |
|  | 1051 | C-C-O (asymmetric stretching) | DEG |
|  | 1007, 953 | P-Phenyl (stretching) | MPB |
|  | 892 | C-C-O (symmetric stretching) | DEG |
|  | 866 | P-CH_3_ (C-H rocking) | MPB |
|  | 615 | O-H (Out-of-plane bending) | DEG |
| 13 | 3318 | O-H (stretching) | TEG |
|  | 2868 | sp^3^ hybridized C–H (stretching) | TEG |
|  | 1486 | =C-H and ring C=C (stretching) | MPB |
|  | 1454 | CH_2_ (bending) | TEG |
|  | 1325 | O-H (In-plane bending) | TEG |
|  | 1325, 1290, 1246 | C–O–H (bending) | TEG |
|  | 1116 | C–O (stretching) in 1° alcohol | TEG |
|  | 1058 | C-C-O (asymmetric stretching) | TEG |
|  | 952 | P-Phenyl (stretching) | MPB |
|  | 885 | C-C-O (symmetric stretching) | TEG |
| 14 | 3300 | O-H (stretching) | W |
|  | 3029 | CH_3_ (asymmetric stretching) / sp^3^ hybridized C–H (stretching) | ChCl |
|  | 2913 | CH of C_1_ (antisymmetric stretching) | Glu |
|  | 1638 | O-H Scissors | W |
|  | 1480 | CH_2_ (bending) | ChCl |
|  | 1031 | υ CO (stretching) | Glu |
|  | 924 | CO (stretching) + CCH (stretching) + ring of pyranose (antisymmetric stretching) | Glu |
|  | 865 | C–N^+^ (symmetric stretching) | ChCl |
|  | 769 | CCO (in-plane bending) + CCH (in-plane bending) | Glu |
| 15 | 3325 | O-H (stretching) | Fru / W |
|  | 2936 | CH of C_2_ (symmetric stretching) | Fru |
|  | 1638 | O-H Scissors | W |
|  | 1478 | CH_2_ (bending) | ChCl |
|  | 923 | CO (stretching) + CCH (stretching) + ring of pyranose (antisymmetric stretching) | Fru |
|  | 865 | C–N^+^ (symmetric stretching) | ChCl / Fru |
|  | 818 | CH (in-plane bending) | Fru |
|  | 781 | CCO (in-plane bending) + CCH (in-plane bending) | Fru |
| 16 | 3293 | O-H (stretching) | W |
|  | 3027 | CH_3_ (symmetric stretching) / sp^3^ hybridized C–H (stretching) | ChCl |
|  | 2927 | CH of C_2_ (symmetric stretching) | Suc |
|  | 1638 | O-H Scissors | W |
|  | 1477 | CH_2_ (bending) | ChCl |
|  | 1050 | CO (stretching) | Suc |
|  | 1001 | CO (stretching) + CCO (in-plane bending) | Suc |
|  | 924 | CO (stretching) + CCH (stretching) + ring of pyranose (antisymmetric stretching) | Suc |
|  | 866 | C–N^+^ (symmetric stretching) / CH (in-plane bending) + CC (stretching) + CC (in-plane bending) | ChCl / Suc |
| 17 | 3304 | O-H (stretching) | W / Gly |
|  | 2936, 2880 | sp^3^ hybridized C–H (stretching) | Gly |
|  | 1650 | O-H Scissors | W |
|  | 1477 | CH_2_ (bending) | ChCl |
|  | 1210 | C–O–H (bending) | Gly |
|  | 1109 | C–O (stretching) in 1° alcohol | Gly |
|  | 1039 | C-C-O (asymmetric stretching) | Gly |
|  | 855 | C-C-O (symmetric stretching) | Gly |
|  | 552 | O-H (Out-of-plane bending) | Gly |
| 18 | 3297 | N-H (stretching) / O-H (stretching) | ChCl |
|  | 2912 | O-H (stretching) | MA |
|  | 1718 | C=O (stretching) | MA |
|  | 1480 | CH_2_ (bending) | ChCl |
|  | 1414 | O-H (In-plane bending) | MA |
|  | 1151 | C-O (stretching) | MA |
|  | 870 | C–N^+^ (symmetric stretching) | ChCl |
