# Supplementary material for: Functionalization of graphene using deep eutectic solvents
Source: Nanoscale Res Lett. 2015 Aug 12;10:324. doi: 10.1186/s11671-015-1004-2 (PMC4531886; doi:10.1186/s11671-015-1004-2)
Supplement: Additional file 5: Table S21. — Description of data: Extracted information from Raman spectra of pristine, oxidized and DES-functionalized graphene oxide. [file 11671_2015_1004_MOESM5_ESM.docx]

Table S.21 Extracted information from Raman spectra of pristine, oxidized and DES-functionalized graphene oxide.

| **Sample** | **G' band** | | **G band** | | **D band** | | **I_D_/I_G_** | **Overlapping peaks in G'** ^d^ |
| --- | --- | --- | --- | --- | --- | --- | --- | --- |
|  | **P (cm^-1^)**^a^ | **I**^b^ | **P (cm^-1^)**^a^ | **I**^b^ | **P (cm^-1^)**^a^ | **I**^b^ |  |  |
| **p-Gr** | 2716.78 | 537.10 | 1575.69 | 1295.13 | 1349.56 | 195.81 | 0.15 | + |
| **o-Gr** | 2705.97 | 1133.41 | 1576.70 | 1728.91 | 1350.07 | 341.24 | 0.20 | - |
| **Gr 1** | 2720.36 | 783.92 | 1576.68 | 981.97 | 1351.21 | 202.70 | 0.21 | + |
| **Gr 2** | 2716.68 | 703.64 | 1579.63 | 1031.16 | 1354.69 | 92.08 | 0.09 | + |
| **Gr 3** | 2715.74 | 769.78 | 1578.37 | 1174.56 | 1355.80 | 104.73 | 0.09 | + |
| **Gr 4** | 2722.13 | 258.62 | 1584.71 | 371.61 | 1350.17 | 143.51 | 0.39 | + |
| **Gr 5** | 2702.52 | 1475.02 | 1570.23 | 1925.82 | 1358.54 | 405.85 | 0.21 | - |
| **Gr 6** | 2719.61 | 651.84 | 1572.35 | 954.22 | 1345.06 | 78.47 | 0.08 | + |
| **Gr 7** | 2702.67 | 1086.15 | 1569.12 | 1721.83 | 1344.78 | 255.45 | 0.15 | - |
| **Gr 8** | 2720.99 | 575.23 | 1581.76 | 1130.35 | 1352.30 | 344.87 | 0.31 | + |
| **Gr 9** | 2716.23 | 1109.91 | 1578.79 | 1629.43 | 1347.68 | 272.72 | 0.17 | + |
| **Gr 10** | 2717.17 | 1260.55 | 1581.54 | 1840.01 | 1351.11 | 462.41 | 0.25 | - |
| **Gr 11** | 2698.62 | 942.64 | 1568.73 | 1916.87 | 1345.04 | 408.08 | 0.21 | + |
| **Gr 12** | 2707.74 | 535.30 | 1573.83 | 1023.47 | 1350.84 | 115.27 | 0.11 | + |
| **Gr 13** | 2708.47 | 646.69 | 1574.72 | 1579.45 | 1346.06 | 500.02 | 0.32 | + |
| **Gr 14** | 2707.01 | 1470.72 | 1572.18 | 1829.47 | 1344.06 | 125.86 | 0.07 | + |
| **Gr 15** | 2721.74 | 638.67 | 1583.95 | 1092.38 | 1348.91 | 360.49 | 0.33 | + |
| **Gr 16** | 2704.45 | 740.56 | 1573.47 | 1155.18 | 1346.30 | 245.62 | 0.21 | - |
| **Gr 17** | 2695.17 | 3341.46 | 1564.83 | 5402.75 | 1343.88 | 159.13 | 0.03 | - |
| **Gr 18** | 2705.63 | 1007.04 | 1573.59 | 1610.24 | 1347.49 | 825.24 | 0.51 | - |
| a: Position of Rama peak, b: Intensity of Raman peak, c: (+) for overlapped peaks in G′ and (-) for sharp peaks | | | | | | | | |
